# Prevalence and awareness of chronic kidney disease among adult diabetic outpatients in Northeast Ethiopia

**DOI:** 10.1186/s12882-020-01768-y

**Published:** 2020-04-15

**Authors:** Temesgen Fiseha, Zemenu Tamir

**Affiliations:** 1 Department of Clinical Laboratory Science, College of Medicine and Health Sciences, Wollo University, Dessie, Ethiopia; 2 Department of Medical Laboratory Sciences, College of Health Sciences, Addis Ababa University, Addis Ababa, Ethiopia

**Keywords:** Chronic kidney disease, CKD awareness, Estimated glomerular filtration rate, Diabetes

## Abstract

**Background:**

Chronic kidney disease (CKD) is a serious complication of diabetes associated with adverse outcomes of renal failure, cardiovascular disease and mortality. Despite this, data regarding the burden and awareness of CKD among adults with diabetes in Sub-Saharan Africa countries are lacking. The aim of this study was, therefore to determine the prevalence and awareness of CKD among diabetic outpatients attending a hospital in Northeast Ethiopia.

**Methods:**

We conducted a cross-sectional study on 323 diabetic adults at the diabetes clinic of a hospital in Northeast Ethiopia, from February 1 to July 30, 2016. Each patient provided a blood sample for serum creatinine and urine for albuminuria. Glomerular filtration rate (eGFR) was estimated using the Modification of Diet in Renal Disease (MDRD) equation. CKD was defined as eGFR < 60 ml/min/1.73 m^2^ and/or albuminuria. Awareness was defined as a positive response to “Has a doctor or other health care professional ever told you that you had kidney disease?”

**Results:**

Of the 323 patients, 85 (26.3%) had Stage 1–5 CKD, 42 (13.0%) had eGFR < 60 ml/min/1.73m^2^ and 58 (18.0%) had albuminuria. In patients with eGFR < 60 ml/min/1.73m^2^ (stage 3–5 CKD), serum creatinine was abnormal (> 1.5 mg/dl) in 23.5% and albuminuria was absent in 31.8%. Of the patients with CKD, only 10.6% of them were aware of their CKD. The proportion of patients who were aware of their disease increased with worsening of CKD stages, from 3.4% of with stage 1 to 75.0% with stage 4. Awareness for all individuals with advanced stages of CKD was only 11.9%. Having albuminuria, high serum creatinine, a family history of kidney disease and being obese were significantly associated with CKD awareness.

**Conclusion:**

A high prevalence but low awareness of CKD was found in diabetic outpatients attending our clinic in Northeast Ethiopia. Our results highlight the need for more diagnostic strategies for CKD screening among diabetic adults and primary care education on the impact of detecting CKD in the early stage to prevent adverse outcomes and improve diabetes care.

## Background

Chronic Kidney Disease (CKD) is a global public health problem associated with an increased risk of end stage renal disease (ESRD), cardiovascular disease (CVD) and premature mortality [1]. It is estimated to affect 8 to 16% of the adult populations of the world, and most of the global burden is occurring in low- and middle-income countries, leading to significant morbidity and mortality due to undertreated CVD and kidney failure [2, 3].

CKD is a well recognised and serious complication of diabetes and diabetes is one of the most common causes of CKD, with up to 44% of patients with CKD affected by diabetes [4]. The presence of CKD in diabetes is associated with an increased risk of CVD, all cause and cardiovascular mortality, and kidney failure; requiring costly renal replacement therapy [5, 6]. It is also associated with significantly higher total all-cause health care costs and costs directly related to treatment of CKD in diabetes [7, 8]. Moreover, due to the rapidly rising incidence of diabetes, the prevalence of CKD as well as the already high medical costs of renal therapy in patients with diabetes are expected to rise further, adding to the global burden of disease and health-care expenditure [9, 10].

Although CKD is common in diabetes and caries a high risk of adverse outcomes, it is often unrecognized; up to 77% of patients receiving diabetes management may have undiagnosed CKD [11, 12]. In addition, because CKD is usually silent until advanced stages, many people with CKD are unaware of its presence and it is identified in its later stages, when opportunities to prevent adverse outcomes are limited [13, 14]. Detection of CKD during its early stages provides opportunity for therapeutic interventions to prevent or delay the onset of complications and improve outcomes [15]. And since lifestyle factors influence the risk of CKD; disease awareness and education on risk are essential for optimal management to prevent or reduce the progression of early CKD, cardiovascular events and mortality [16–18].

Despite existing evidence suggesting that CKD is a substantial health burden among diabetic patients on the African continent [19], data regarding the burden and awareness of CKD among adults with diabetes in Sub-Saharan Africa are lacking. Therefore, in this study we aimed to determine the prevalence and awareness of CKD among diabetic adult outpatients attending a hospital in Northeast Ethiopia.

## Methods

### Study setting and population

This cross-sectional study was conducted from February 1 to July 30, 2016 at the outpatient diabetes clinic of Dessie Referral Hospital (DRH), Northeast Ethiopia. The Hospital is found in Dessie town of Amhara regional state and serves as a referral center for South Wollo and surrounding zones. It delivers diagnostic, therapeutic and monitoring services to all diabetic patients and also tertiary diabetes patient care. Adult patients (aged ≥18 years) already diagnosed with diabetes mellitus attending the follow-up diabetes clinic of the hospital during the study period were included in the study. Diabetic patients who were hospitalized, pregnant (woman), with acute illness (fever), underwent treatment with dialysis and not fasting were excluded from the study. The study protocol was approved by the Institutional Review Board and Research Ethics Committee of Wollo University, College of Medicine and Health Sciences. An informed written consent was obtained from all the participants enrolled.

### Sample size

The sample size calculation was based on an estimated prevalence of CKD of 50% in that population, with a confidence level of 95 and 5% precision. A sample size of at least 317 individuals was needed to be representative of the 1800 individuals on diabetic follow-up in our site to obtain 80% power. Considering a 5% non-response rate, the sample size was increased to 333 patients. Estimates of CKD prevalence were obtained on the 323 participants. Systematic random sampling was used to select study participants.

### Data collection and laboratory measurements

Data on socio-demographic, medical and family history were collected by using a pre-tested structured interviewer administered questionnaire (see supplementary file 1). Participants who completed the interview process had their anthropometric data such as weight (kilograms), height (meters), and blood pressure (mm Hg) collected. Body mass index (BMI) was calculated as the ratio of the weight (kg) to the square of the height (meters) and classified as: normal/underweight (BMI < 25 kg/m^2^), overweight (BMI 25–29.9 kg/m^2^) and obese (BMI ≥30 kg/m^2^). Blood pressure (BP) was measured using a manual mercury sphygmomanometer, with the patient remaining in a sitting position for at least 15 min before the first measurement. Each participant’s blood pressure was measured three times at an interval of at least 5 min, and then averaged to be recorded. Hypertension was defined as systolic BP ≥140 mmHg and/or diastolic BP ≥90 mmHg or current use of antihypertensive medication. Awareness of kidney disease was ascertained by asking the question “Have you ever been told that you had a kidney disease by a doctor or other health care professional?” Do not include kidney stones or bladder infection.

Blood samples were drawn early in the morning after an overnight fasting and biochemical analyses were performed on Dirui CS-T240 clinical chemistry analyzer (Dirui Industrial Company, China) using kits supplied by the manufacturer. Blood glucose was measured by enzymatic GOD-PAP method. Serum creatinine was measured using a Jaffe kinetic method, with calibration traceable to IDMS reference material. Fresh urine sample was collected from each participant to detect albuminuria using dipsticks (COMBINA 11S, Human). Presence of albuminuria (+ 1 or more) in urine was defined as albuminuria.

The four-variable Modification of Diet in Renal Disease (MDRD) study equation was used to estimate glomerular filtration rate (eGFR) [20]. CKD was defined as eGFR < 60 ml/min/1.73 m^2^ and/or albuminuria on two occasions spaced by 2 weeks. Stages of CKD were classified according to the National Kidney Foundation Kidney Disease Outcomes Quality Initiative (NKF KDOQI) guideline as [21]: stage 1, eGFR ≥90 ml/min/1.73 m^2^ with albuminuria; stage 2, eGFR of 60–89.9 ml/min/1.73 m^2^ with albuminuria, and stages 3, 4 and 5 as eGFR of 30–59.9, 15–29.9 and < 15 ml/min/1.73 m^2^, respectively. Stage 3 was further classified into 3A (45–59.9 ml/min/1.73 m^2^) and 3B (30–44.9 ml/min/1.73 m^2^) [22].

### Statistical analysis

Data were entered into “EpiData 3.1” software and analyzed using SPSS version 20.0 (SPSS, Chicago, IL, USA). Data were expressed as means ± standard deviation (SD) or percentage. Comparisons between groups were done by chi square (x^2^) test or *t*-test as appropriate. Multivariable stepwise logistic regression was used to calculate adjusted odds ratios (OR) and the corresponding 95% confidence intervals (CI). *P* value < 0.05 was used to indicate statistical significance.

## Results

### Demographic and clinical characteristics of participants

A total of 323 patients were included in the study. The mean (± SD) age of the participants was 44 ± 15.7 years (ranging from 18 to 77 years), and 168 (52.0%) were males. Of the participants, 173 (53.6%) were type 2 diabetics, 186 (57.6%) had duration of diabetes less than 5 years, and 62 (19.2%) had a family history of kidney disease. Mean BMI was 22.37 ± 3.94 Kg/m^2^. The mean systolic and diastolic BP of the participants was 125.6 ± 18 and 78.4 ± 8 mmHg, respectively. Mean FBG was 190.15 ± 87.89 mg/dl. Mean SCr was 1.01 ± 0.33 mg/dl. The mean eGFR was 100.6 ± 35.04 ml/min/1.73 m^2^ (Table 1).

**Table 1:** Demographic and clinical characteristics of study participants (*n* = 323)

Characteristics	Category	N (%)
Age (year), means ± SD		44 ± 15.7
Age group, n (%)	18–39 Years	119 (36.8)
	40–49 Years	75 (23.2)
	50–59 Years	62 (19.2)
	60–69 Years	48 (14.9)
	≥70 Years	19 (5.9)
Sex, n (%)	Male	155 (48.0)
	Female	168 (52.0)
Type of diabetes, n (%)	Type 1	150 (46.4)
	Type 2	173 (53.6)
Duration of diabetes, n (%)	< 5 Years	186 (57.6)
	5–9.9 Years	97 (30.0)
	≥10 Years	40 (12.4)
Family history of kidney disease, n (%)		62 (19.2)
Body mass index (Kg/m^2^), means ± SD		22.37 ± 3.94
Obesity, n (%)		21 (6.5%)
Hypertension, n (%)		99 (30.7%)
Systolic BP (mmHg), means ± SD		125.6 ± 18
Diastolic BP (mmHg), means ± SD		78.4 ± 81
Fasting Serum Glucose (mg/dl), means ± SD		190.15 ± 87.89
Serum Creatinine (mg/dl), means ± SD		1.01 ± 0.33
eGFR _MDRD_ (ml/min/1.73 m^2^), means ± SD)		100.6 ± 35.04

### Prevalence of CKD

The overall prevalence of CKD stage 1–5 was 26.3% (95% CI, 20.8 to 31.8) (Table 2). Of the total participants, 13.0% had an eGFR of < 60 ml/min/1.73m^2^ (stage 3–5 CKD). Only 23.5% of the participants with advanced stage CKD had SCr values > 1.5 mg/dl (the traditional criteria for diagnosing CKD; eGFR < 60 ml/min/1.73m^2^) [23]. Albuminuria was present in 18% of total participants, but was absent in 31.8% of those with eGFR < 60 ml/min/1.73m^2^ (stage 3–5 CKD). The prevalence of CKD was 46.9% in participants > 60 years old and 22.6% in those ≤60 years old (*P* < 0.001). CKD prevalence was lower in males (14.8%) than in females (36.9%; *P* < 0.001).

**Table 2:** Prevalence of CKD stages according to K/DOQI classification (*N* = 323)

CKD Stage	eGFR (ml/min/1.73m^**2**^)	Number (%)
1	≥90	29 (9.0)
2	60–89.9	14 (4.3)
3	30–59.9	38 (11.8)
3A	45–59.9	33 (10.2)
3B	30–44.9	5 (1.6)
4	15–29.9	4 (1.2)
All stages of CKD		85 (26.3)
Albuminuria		58 (18.0)

In univariate analysis, the factors associated with CKD were older age (*P* < 0.001), female gender (*P* < 0.001), decreased literacy (*P* = 0.017), low monthly income (*P* = 0.042), long duration of diabetes (*P* < 0.001), family history of kidney disease (*P* < 0.001), hypertension (*P* < 0.001), high systolic BP (*P* = 0.005), and poor glycemic control (*P* < 0.001). In the multivariable analysis, older age (OR = 2.48, CI 1.13–5.43), gender (OR = 4.93, CI 2.56–9.50), longer duration of diabetes (OR = 5.16, CI 2.13–12.51), hypertension (OR = 3.37, CI 1.45–7.86), a family history of kidney disease (OR = 2.37, CI 1.17–4.79), and poor glycemic control (OR 2.58, CI 1.19–5.61) were significantly associated with CKD (Table 3).

**Table 3:** Multivariate logistic regression analysis of risk factors for CKD in our cohort of diabetic patients

Variable	Odds ratio	95% Confidence interval	*P*-value
Age > 60 years	2.48	1.13–5.43	0.023
Female gender	4.93	2.56–9.50	0.001
Diabetes duration ≥10 years	5.16	2.13–12.51	< 0.001
Family history of kidney disease	2.37	1.17–4.79	0.016
Hypertension	3.37	1.45–7.86	0.005
Mean fasting blood glucose > 130 mg/dl	2.58	1.19–5.61	0.016

### CKD awareness

Overall, 10.6% of 85 individuals with CKD reported being told by a doctor or other health care professional that they had kidney disease. The proportion of individuals who were aware of their disease increased with worsening of CKD stages: 3.4% of with stage 1, 21.4% with stage 2, 5.3% with stage 3, and 75.0% with stage 4. Awareness for all individuals with advanced stages of CKD (stage 3–5 CKD) was only 11.9%. For logistic regression, CKD awareness was used as the outcome variable. The covariates were age, sex, duration of diabetes, family history of kidney disease, obesity, serum creatinine and albuminuria. In multivariable analysis, having albuminuria (OR = 4.08, 1.54–10.85), abnormal serum creatinine (OR = 5.16, 1.52–17.48), and a family history of kidney disease (OR = 2.89, 1.07–7.80) and being obese (OR = 5.53, 2.18–14.04) were all statistically significantly associated with greater awareness (Table 4).

**Table 4:** Factors associated with CKD awareness

Variable	Odds ratio	95% Confidence interval	*P* value
Albuminuria	4.08	1.54–10.85	0.017
Serum creatinine > 1.5 mg/dl	5.16	1.52–17.48	0.001
Family history of kidney disease	2.89	1.07–7.80	0.002
Obesity (BMI ≥30 kg/m^2^)	5.53	2.18–14.04	0.042

## Discussion

The prevalence of CKD of any stage [1–5] in our diabetic outpatients was 26.3%, with stage 3 being dominant (11.8%). The overall prevalence of CKD observed in our study was higher than 21.8% prevalence reported from the University of Gondar Hospital study in Northwest Ethiopia [24] and closer to that of 27.5% reported in the Netherland study [25], 27.9% in the Spain PERCEDIME2 study [26] and 29.6% in the Chinese study [27]. However, our prevalence estimate of CKD using a similar definition of eGFR prediction equation was lower than that of 39% reported in Kinshasa, the democratic republic of Congo study [28], 34.1% in Catalonia (Spain) study [29], and 42.3% in the Japanese study [30]. These differences in prevalence might be because of the differences in creatinine assays and calibration, albumin assays or differences in case-mix (in terms of diabetes type).

A high prevalence of advanced stages CKD (defined as eGFR < 60 ml/min/1.73 m^2^; stages 3–5 CKD), 13.0% was found in our studied diabetic outpatients using eGFR equations. This is in accordance with other studies that found a prevalence of 3.9–31% of eGFR _MDRD_ < 60 ml/min/1.73 m^2^ [11, 24, 31]. Interestingly, only 23.5% of those with advanced stages CKD had serum creatinine level > 1.5 mg/dl, the point frequently used by clinicians for diagnosing CKD (eGFR < 60 ml/min/1.73m^2^; stages 3–5 CKD). This is in accordance with our previous study, reporting only 48.7% of diabetic outpatients with eGFR _MDRD_ < 60 ml/min/1.73 m^2^ had abnormal serum creatinine values [32]. We also found that albuminuria was absent in 31.8% of subjects with CKD stages 3 to 5 (eGFR < 60 ml/min/1.73m^2^). In line with this a recent study found that approximately one half (46.6%) of diabetics with CKD had reduced eGFR without albuminuria [33]. Inadequacies of CKD diagnosis in diabetes from routine clinical assessment also are mentioned frequently in the literature [11, 12, 31], indicating the need for a simple functional measurement of GFR, as provided using the eGFR.

Our data shows that CKD awareness rates in diabetic outpatients are low: 3.4% for people with stage 1 CKD, 21.4% for stage 2, 5.3% for stage 3, and 75.0% for stage 4. These findings complement with the ADD-CKD Study [13], in which 1.1% of diabetics with Stage 1, 4.9% with Stage 2, 18.0% with Stage 3, 52.9% with Stage 4 were aware of their CKD. Awareness among KEEP participants with diabetes and CKD was low at 9.4%, being 6% in patients with CKD stages 1 and 2, and 10.9% in those with advanced stages of CKD [34]. Our finding of higher CKD awareness in patients with a family history of renal disease is supported by the KEEP data, which suggested that a family history of kidney disease should make CKD awareness more likely [35]. The low CKD awareness, which is magnified by the use of inadequate screening tests such as serum creatinine and dipstick albuminuria in this study, indicates that primary care physicians may not be able to identify diabetics with CKD based on the laboratory report in hand [13]. Such a finding therefore indicates the need of routine use of eGFR to improve the recognition and thus, awareness of CKD in primary care.

Currently, most laboratories in Ethiopia do not report an eGFR when renal profile or serum creatinine is ordered. As blood test for serum creatinine or urine dipstick for albuminuria are used by clinicians to determine the presence or absence of kidney disease, this form of screening can lead to underrecognition of CKD [11, 31] and thus low disease awareness [36]. Early detection and awareness of CKD in diabetics can therefore be improved by routine eGFR reporting and primary care education on the impact of introducing eGFR into the routine screening of CKD to facilitate its early recognition and the institution of effective preventive measures for modifying disease outcomes and improving diabetes care, as shown by related studies [37–39]. Supporting this, a recent study noted that determination of the eGFR of even newly diagnosed asymptomatic diabetics with a view to creating awareness for early screening, evaluation and intervention would be desirable [40]. GFR can be estimated by the Cockcroft and Gault [41] or the MDRD study equations, as indicated by the NKF and the American Diabetes Association [42].

The 4-variable MDRD study equation was used for estimating GFR in this study, as recommended by the NKF KDOQI guidelines, as it generates a GFR estimation normalized to a standard body surface area (1.73 m^2^) using sex, age, race and serum creatinine only. Because it is more accurate than the Cockcroft-Gault equation in CKD patients with diabetes [43], and is not biased by body weight [44] and more robust when glucose control is poor [45]; the MDRD equation appears to be preferable. Additionally, the routine laboratory estimation of GFR using the simplified MDRD equation has been also shown to facilitate the recognition and documentation of early CKD and increase awareness of CKD in such patients with diabetes [37, 38]. There is also evidence that the estimation of early GFR loss is more accurate with the MDRD equation than with the recently developed Chronic Kidney Disease Epidemiology (CKD–EPI) equation in diabetic population [46]. Further studies are needed to investigate whether eGFR reporting with the MDRD equation facilitates the recognition and awareness of CKD in Ethiopian diabetic adults.

This study was the first of its type to determine the prevalence and awareness of CKD among African diabetic adult outpatients but has some limitations. The study’s limitations include the small sample size of our study population and the fact that data was taken from a single hospital, thus our sample cannot be considered representative of all diabetic patients in the country. On the other hand, more than half (57.6%) of the patients had a short duration of diabetes, and this may explain some of the differences with other studies. We used the MDRD study equation, the validation of which is lacking among Ethiopian diabetic adults. The CKD-EPI equation may be superior in estimating the GFR but the best eGFR equation for Ethiopian adults has not yet been determined. Finally, the measurement of serum creatinine was not standardized; this might influence the performance of eGFR equations.

## Conclusion

In conclusion, this study demonstrated a high prevalence and low awareness of CKD among diabetic adults attending the outpatient diabetes clinic of Northeast Ethiopia. Current screening methods, serum creatinine or urine dipstick for albuminuria, underestimates the presence of clinically significant CKD in our diabetic outpatient. More diagnostic strategies for CKD screening among diabetic adults and primary care education on the impact of detecting CKD in the early stage are needed to prevent adverse outcomes and improve diabetes care.

## Supplementary information

**Additional file 1:.** Questionnaire. English version of the questionnaire used during the study.

## Data Availability

The data of this study can’t be shared publicly due to presence of sensitive (confidential) participants’ information and additional data than that used in this publication. But the data are available from the corresponding author on reasonable request.

## Abbreviations

BMI: Body mass index
BP: Blood pressure
CKD: Chronic kidney disease
DRH: Dessie Referral hospital
eGFR: Estimated glomerular filtration rate
