# Supplementary material for: Prevalence and awareness of chronic kidney disease among adult diabetic outpatients in Northeast Ethiopia
Source: BMC Nephrol. 2020 Apr 15;21:129. doi: 10.1186/s12882-020-01768-y (PMC7161144; doi:10.1186/s12882-020-01768-y)
Supplement: Supplementary file 1 — Additional file 1:. Questionnaire. English version of the questionnaire used during the study. [file 12882_2020_1768_MOESM1_ESM.docx]

**Questionnaire: English version of the qquestionnaire used during the study on Prevalence and awareness of chronic kidney disease among adult diabetic outpatients in Northeast Ethiopia**

| PART 1- Questions Related to Socio-Demographic Characteristics  Identification No.__________________ | | |
| --- | --- | --- |
| \| Q. \|  \|  \| \| --- \| --- \| --- \| | QUESTION | RESPONSE CODE (Check the Appropriate Option or Enter Numbers) |
| 100 | What is the sex of the respondent? | 1. Male  2. Female |
| 101 | What is the age of the respondent? | Age in years (18-99) ________ (yy.m)  (write “99+”, if >99 years) |
| 105 | What is the educational status of the respondent? | \| 1. Illiterate  2. Read & write  3. Grade1-8 \| 4. Grade9-12  5. College & above \| \| --- \| --- \| |
| 106 | Annual income of the Respondent | _____________ ETB |
| 107 | Have you ever smoke cigarette in the past 12 month? | 1.Yes  2. No |
| 108 | Have you ever been told that you had a kidney disease by a doctor or other health care professional?” Do not include kidney stones or bladder infection | 1.Yes  2. No |
| PART 2: Clinical and Laboratory Measurements | | |
| 200 | Types of diabetes | \| 1. Type 1  2. Type 2 \|  \| \| --- \| --- \| |
| 201 | Duration of disease. | \| 1. < 5 years  2. 5-9.9 years  3. ≥ 10years \|  \| \| --- \| --- \| |
| 202 | Family History of kidney disease | 1.Yes  2. No |
| 203 | Current blood Pressure measurement (Systolic/Diastolic) | 1^st^.______/____ mmHg  2^nd^.______/____ mmHg  3^rd^.______/____ mmHg  Average_______/______ mmHg |
| 204 | Weight | _____________kg (kilogram) |
| 205 | Height | ____________m (meter) |
| 206 | Blood glucose level | ___________mg/dl |
| 207 | Serum creatinine level | ___________mg/dl |
| 208 | Albuminuria | 1.Present  2.Absent |
